# Medical career expectations of academically talented high school students: a nationwide cross-sectional study in China

**DOI:** 10.1186/s12909-020-02083-8

**Published:** 2020-05-24

**Authors:** Hongbin Wu, Leisi Pei, Shan Li, Cheng Jiang

**Affiliations:** 1 Institute of Medical Education/National center for Health Professions Education Development, Peking University, Beijing, China; 2 Faculty of Education, The University of Hong Kong, Hong Kong SAR, China; 3 Department of Educational and Counselling Psychology, McGill University, Montreal, Canada; 4 Peking University, Graduate School of Education, No.5 Yiheyuan Road, Haidian District, Beijing, 100871 China

**Keywords:** Career expectations, Medical career, Talented students, High school students, China

## Abstract

**Background:**

Academically talented high school students (ATHSSs), an exceptional cohort, are not well studied for their career expectations, especially for those with medical career expectation (MCE). Nowadays, the public perception of the medical profession is changing in China. The purpose of this study was to answer questions about ‘is medicine attractive for ATHSSs and ‘what factors affect medical career expectations (MCE) for ATHSSs’ in China.

**Methods:**

A total of 16,479 representative ATHSSs in senior three completed a questionnaire and four different academic tests. Frequency statistics showed the proportion of ATHSSs with MCE. Unpaired t-tests were performed to find out the differences in demographics, family background, and academic performance between students with and without MCE. The logit models analysis were applied to explore the potential factors that affected the MCE of this exceptional group of students.

**Results:**

ATHSSs with MCE accounted for 20.6% (ranking 7/18) of the respondents. They were more likely to be female, came from relatively poorer families, lived in a rural area, and performed significantly worse in all academic tests except for mathematics, compared with those without MCE. In addition, the results revealed that gender (β = − 0.436, *p* < 0.01), region of hometown (β = − 103, *p* < 0.1), mother’s years of schooling (β = − 0.019, *p* < 0.05), and father’s occupational status (β = − 0.005, *p* < 0.01) contributed significantly to the MCE of academically talented students. Better performance in mathematics affected the MCE of ATHSSs taking the liberal arts and science tests differently.

**Conclusions:**

We found the medical career is becoming unattractive to academically talented students and the medical career may be losing their aura in China. Students who have medical career expectations are likely to be females and to have a weak family background.

## Background

Career expectations, can be defined as the occupation students expected to pursue [1], and are jointly influenced by personal and environmental factors [2]. The career expectations of general high school students and adolescents have been extensively studied over decades, accumulating informative evidence for educational policies. Previous studies have formed coherent conclusions that demographic profiles such as gender [3], family background [4], self-belief/efficacy [5], social support [6, 7] (e.g. support from parents, teachers and peers) and cultural context [8] have a significant influence on students’ career choices. Nevertheless, academically talented high school students (ATHSSs), an exceptional cohort that tends to be more accomplished and influential in their career development and thus to impact the distribution of future elites in various fields [9], have not been well studied in terms of their career expectations, especially those with medical career expectations (MCE) .

The health profession, which is an extremely demanding career, provides a critical safeguard for human health and safety. It is rather competitive and selective in general, and qualified students compete for admission to higher medical education institutions across the world [10]. A review of the literature revealed that when it comes to examining MCE, most studies have focused on medical students who have already been enrolled in colleges and universities. To be specific, a wide range of research on the career trajectory of health professionals after graduation has explored how different factors [11–13] (e.g. gender, family background, indebtedness and academic performance) affect medical students’ choices of work location (urban or rural) [14, 15], work status (full-time or part-time) [16], profession (surgeon, physician, general practitioner, etc.) [17], career success and drop-out rates [18].

In this study, we are particularly interested in ATHSSs for the following reasons. First, research has shown that academically talented students tend to follow a career path set in their adolescence and they are far less affected by the factors from the broader social environment due to their self-efficacy [19]. Second, this exceptional cohort may undertake extra challenges in furtherance of their career decision-making that their peers do not attempt. This requires more attention from researchers. Third, medicine is highly demanding, competitive and selective in its nature. ATHSSs are potentially qualified to compete for admission to higher medical education institutions across the world. Finally, the public perception of the medical profession is changing in China, due to worsening doctor–patient relationships and the stagnant salaries paid to physicians [20]. It is thus timely to consider whether medicine is still attractive to ATHSSs and what factors affect the choice of a medical career among ATHSSs.

To this end, two research questions (RQs) are raised in this exploratory study: RQ1, what is the current status of ATHSSs’ MCE across China compared with other careers; and RQ2, what factors affect the MCE of ATHSSs across China? Based on the aforementioned discussion, we considered students’ demographics, family background and academic performance. The findings from this study not only have the potential to generate theoretical knowledge but also to inform policy-making for cultivating medical talent. It is important to note that MCE covers not only hospital professionals but also medical scientists doing research in the lab. Among these various professions, ‘doctor’ is the most commonly used term among the public, especially in the Chinese culture. Therefore, the term ‘doctor’ in this study represents MCE when investigating the career expectations of ATHSSs.

## Methods

### Participants

This study is part of the ‘Assessment of Core Competencies (ACC)’ Project. ACC project is a large-scale pioneering research project launched in 2017 by Graduate School of Education in Peking University. The ACC project aims to assess the core competencies of high school students in China and help them transit smoothly to college studies. This project brings together researchers and educators from over 60 top senior highs and more than 10 educational research institutes in top universities across China. To identify ATHSSs, 265 top senior high schools, with obvious superiority in student learning achievement, across 26 Chinese provinces (including municipalities and autonomous regions) were selected for the first step. One of the criteria for choosing top senior high schools was that the average admission rate into first-tier universities. Specifically, the average admission rate among the senior high schools sampled was more than three times the national average in 2017. In addition, the average admission rate into the top universities (i.e. Peking University and Tsinghua University) was about 10 times the national average. In each of the 265 high schools, academically talented students were identified as those with the greatest possibility of being accepted by a first-tier (key) university based on their prior academic performance. This resulted in a total of 18,618 students. All of the students in this study were senior three students with an average age of 17, who would take the National College Entrance Examination (NCEE) and choose a major to study at university in June 2018. Like other majors, students are enrolled to medical schools on the basis of whether or not choosing the related majors and scores of NCEE.

The ATHSSs were asked to complete a questionnaire pertaining to their demographics (i.e. gender), family background (i.e. parents’ socioeconomic status, education, family income, region of their hometown) and career expectations from 2nd December to 13th December 2017. In addition, they were required to take three shared tests in Chinese language and literature, mathematics and English on 17th December 2017. In China, there are two academic tracks in high school: liberal arts and science. Those who chose the liberal arts track were asked to complete an additional exam on liberal arts. The group who chose the science track were asked to complete an additional test on science. These tests of academic performance resembled the NCEE, in which questions are assigned by experts from senior high schools and distinguished professors from related fields at Peking University. Chinese students take either the liberal arts test or science test based on their choice of academic track in high school. Among the academically talented students from 242 top senior high schools, 16,479 out of 18,618 completed both the questionnaire and the four academic tests.

### Measures

#### MCE

MCE were obtained from the student survey, in which a question was asked about their expected careers. Prior to the survey, a list of careers was collaboratively created by six specialists, including two high school principals, one experienced high school teacher, and three researchers from Peking University. Eighteen representative careers were presented. ‘Doctor’, which was treated as representative of MCE, was one of the choices. The students were asked to choose three careers. In data analysis, those who choose ‘doctor’ were considered to have MCE. One dummy variable was set to indicate whether the students had MCE, i.e. having MCE = 1 and having no MCE = 0.

#### Demographic characteristics

The students’ demographic characteristics were examined by gender variables. We did not consider an ethnic variable because 95.4% of the students in the sample were Han Chinese. The students’ gender was treated as a dummy variable, with male = 1 and female = 0.

#### Family background

We measured the students’ family background in terms of their parents’ socioeconomic status, educational background, family income and region of their hometown. The parents’ career status was represented by the International Socio-Economic Index (ISEI) based on their career classification [21]. The parents’ education background was indicated by their years of schooling. Family income was determined by the students’ subjective estimation of their family income compared to others in their living area. Specifically, three choices were provided: high, middle and low income. For analysis, two different dummy variables were used to indicate whether students came from middle-income or high-income families. In addition, a dummy variable was created for the region of the hometown, with 1 = urban and 0 = rural.

#### Academic performance

Each of the students took four academic tests. Consistent with the practice of the National College Entrance Examination, we transformed the original scores of each student in the four academic tests into percentile scores.

### Data analysis

We used frequency statistics, unpaired t-tests, and logit models to address our research questions. First, the distribution of the ATHSSs’ career expectations was calculated. Then, the differences in family background and academic performance were examined for the ATHSSs with and without MCE using unpaired t-tests. Finally, logit models was applied to explore the potential factors that might affect the MCE of the ATHSSs. All analyses were conducted using Stata 13.0.

## Results

### Respondents

The questionnaire and tests were successfully completed by 16,479 ATHSSs from 242 top senior high schools across 20 provinces (including municipalities and autonomous regions) resulting in a response rate of 88.50%. For the 242 sample schools in this study, the average admission rate (64.55%) into first-tier universities was almost four times the average admission rate in the 20 provinces. The average number of students enrolled in Peking University and Tsinghua university in 2017 and from 2013 to 2017 were 9.35 and 46.21 respectively, which are both about nine times the national average.

Among the respondents, 26.42% chose the liberal arts academic track, 53.78% were male and 77.67% were from an urban area of China (Table 1).

**Table 1:** Descriptive statistics of the sample

Sampled School (*n* = 242)			Mean	SD
Admission	Enrolment in Peking and Tsinghua University in 2017 (N)		9.35	14.66
	Enrolment in Peking and Tsinghua University during 2013–2017 (N)		46.21	68.40
	Admission rate into first-tier Universities in 2017 (%)		64.55	25.67
Respondents (*n* = 16,479)		N	Percentage (%)	
Major	Liberal Arts	4354	26.42	
	Science	12,125	73.58	
Gender	Male	8862	53.78	
	Female	7617	46.22	
Region	Urban	12,800	77.67	
	Rural	3679	22.33	

*SD* standard deviation

### Current status of medical career expectations of ATHSSs

Table 2 shows the distribution of career expectations among the ATHSSs. From the 18 career choices, ‘economist or financial analyst’ (33.49%) ranked first, followed by ‘college/university faculty’ (33.07%) and ‘freelancer or start-up pioneer’ (32.24%). In contrast, ‘doctor’ only accounted for 20.60% and ranked seventh overall.

**Table 2:** Distribution of ATHSSs’ career expectations (*n* = 16,479, %)

Career expectations	Percent (%)	Rank
Economist or financial analyst	34.49	1
College/university faculty	33.07	2
Freelancer or start-up pioneer	32.24	3
Scientist	31.08	4
Engineer	27.14	5
Computer technician	23.34	6
Doctor	20.60	7
Entrepreneur	17.36	8
Employee of state organs and public institutions	16.13	9
Lawyer	13.38	10
Writer	12.48	11
Journalist	8.71	12
Government official	8.44	13
Poet or artist	8.28	14
Primary and secondary school teacher	7.18	15
Police officer	2.70	16
Actor or sports star	2.38	17
Kindergarten teacher	0.99	18

### Potential factors affecting medical career expectations of ATHSSs

#### Unpaired t-tests

ATHSSs with and without MCE differed significantly in many aspects (Table 3). The ratio of male ATHSSs with MCE was significantly lower than those without MCE (t = 8.52, *p* < 0.01). The proportion of students from urban areas with MCE was significantly lower than those without MCE (t = 7.38, *p* < 0.01).

**Table 3:** Unpaired t-tests of ATHSSs with and without MCE

	Without MCE (*n* = 13,089)		With MCE (*n* = 3390)		Dif	t	p	Cohen’s d
Variable	Mean	SD	Mean	SD				
Male	0.56	0.50	0.47	0.50	0.08	8.52	0.00	0.16
Urban	0.79	0.41	0.73	0.44	0.06	7.38	0.00	0.14
Father’s years of schooling	13.81	3.72	13.25	3.79	0.56	7.77	0.00	0.15
Mother’s years of schooling	13.18	3.86	12.53	3.94	0.65	8.76	0.00	0.17
Father’s ISEI	47.03	21.10	43.15	20.89	3.88	9.55	0.00	0.18
Mother’s ISEI	42.90	20.40	39.81	20.07	3.09	7.88	0.00	0.15
High-income family	0.13	0.33	0.09	0.28	0.04	6.50	0.00	0.13
Middle-income family	0.61	0.49	0.61	0.49	0.01	0.59	0.28	0.01
Score for Chinese	51.46	28.68	47.59	28.66	3.88	7.00	0.00	0.13
Score for mathematics	50.72	29.05	50.37	27.50	0.35	0.63	0.26	0.01
Score for English	51.64	28.63	47.80	28.59	3.84	6.84	0.00	0.13
Score for liberal arts^a^	50.47	23.17	41.98	22.86	8.50	6.50	0.00	0.37
Score for science^a^	51.67	22.87	46.44	22.20	5.23	11.01	0.00	0.23

*SD* standard deviation, *Dif* difference between two groups, *ISEI* the International Socio-Economic Index

^a^Among the ATHSSs without MCE, 4014 took the liberal arts test, and 9075 took the science test; among the ATHSSs with MCE, 340 took the liberal arts test, and 3050 took the science test

Among the ATHSSs with MCE, the parents’ years of schooling were significantly lower than those without MCE (father: t = 7.77, *p* < 0.01; mother: t = 8.76, *p* < 0.01). For parents’ ISEI, there were also significant differences between ATHSSs with and without MCE (father: t = 9.55, *p* < 0.01; mother: t = 7.88, *p* < 0.01). Specifically, both the fathers and mothers of ATHSSs with MCE were significantly lower on the ISEI than those without MCE. In addition, the proportion of ATHSSs with MCE who came from high-income families was significantly lower than those without MCE (t = 6.50, *p* < 0.01). However, no difference was found for middle-income families (t = 0.59, *p* = 0.28).

On academic performance, ATHSSs with MCE performed significantly worse on the academic tests than those without MCE (Chinese: t = 7.00, *p* < 0.01; English: t = 6.84, *p* < 0.01; liberal arts: t = 6.50, *p* < 0.01; science: t = 11.01, *p* < 0.01), with the exception of mathematics (t = 0.63, *p* = 0.26). The biggest gap between ATHSSs with and without MCE was found in the liberal arts, which on average reached 8.49 points.

Considering that *p*-values are affected by sample size in t-tests, we calculated the effect size of Cohen’s d for each t-test to help interpret the importance of differences observed. The results in Table 3 showed that all the significant t-tests were with a small effect size except the t-test for the liberal arts, which had a small to medium effect size.

#### Logit models

Four logit models were conducted to examine the potential factors that affected the ATHSSs’ MCE. ATHSS with MCE was viewed as a dependent variable, and the stepwise method was used to obtain a summary of the results (Table 4). Model 1 controlled the demographic characteristics and family background of the ATHSSs, and model 2 further controlled the factors of academic performance on Chinese, mathematics and English. Considering that the students took either the liberal arts or science test, two separate analyses were carried out as model 3 and model 4.

**Table 4:** Summary results of the Logit model estimations

	Logit model 1	Logit model 2	Logit model 3	Logit model 4
Dependent variable	MCE			
Male	−0.342***	− 0.436***	− 0.681***	− 0.753***
	[0.039]	[0.042]	[0.168]	[0.046]
Urban	−0.126**	−0.103*	− 0.264*	−0.072
	[0.054]	[0.055]	[0.152]	[0.061]
Father’s years of schooling	0.01	0.012	0.009	0.009
	[0.009]	[0.009]	[0.027]	[0.010]
Mother’s years of schooling	−0.021**	− 0.019**	− 0.042	− 0.025**
	[0.009]	[0.009]	[0.027]	[0.010]
Father’s ISEI	−0.006***	−0.005***	− 0.002	−0.006***
	[0.001]	[0.001]	[0.004]	[0.002]
Mother’s ISEI	0.00	0.00	−0.002	0.00
	[0.001]	[0.001]	[0.004]	[0.002]
High-income family	−0.277***	−0.265***	− 0.219	−0.242***
	[0.081]	[0.083]	[0.258]	[0.091]
Middle-income family	0.002	0.014	0.095	0.003
	[0.050]	[0.051]	[0.148]	[0.057]
Score for Chinese		−0.004***	−0.002	− 0.001
		[0.001]	[0.002]	[0.001]
Score for mathematics		0.004***	0.009***	−0.004***
		[0.001]	[0.002]	[0.001]
Score for English		−0.003***	−0.007***	− 0.002*
		[0.001]	[0.002]	[0.001]
Score for liberal arts			−0.013***	
			[0.003]	
Score for science				− 0.003***
				[0.001]
Constant	−0.655***	− 0.525***	− 0.924***	0.396***
	[0.078]	[0.085]	[0.243]	[0.100]
LR chi2(12)	206.34	276.14	102.55	505.54
Prob > chi2	0.000	0.000	0.000	0.000
Pseudo R2	0.013	0.017	0.044	0.039
Observations	16,479	15,794	4209	11,585

Standard errors in brackets; *** *p* < 0.01, ** *p* < 0.05, * *p* < 0.1.

The ATHSSs with MCE were more likely to be female, and this was exacerbated when academic performance was controlled, becoming 35.4% (1 - (e^-0.436^) = 0.354) higher than males. The logit models showed that ATHSSs with MCE were less likely to be from an urban area. Specifically, the percentage of ATHSSs with MCE from urban areas was significantly lower than those from rural areas (1 - (e^-0.103^) = 0.098), when controlling for demographic characteristics, family background and academic performance. However, this difference was more nuanced when the science test score was controlled. The results also showed that father’s years of schooling had no significant impact on ATHSSs’ MCE. In contrast, mother’s years of schooling had a significantly negative effect on MCE. In addition, high-income families showed a remarkably negative attitude towards MCE whereas middle-income families did not.

In terms of the academic tracks of liberal arts and science, the models revealed that both tracks had a significant influence on the MCE of ATHSSs. In general, ATHSSs with MCE did much worse on the liberal arts test compared to the science test. In addition, for every 1-point increase in the liberal arts test, the possibility of an ATHSS having MCE decreased by 1.3% (1-e^-0.013^). However, the higher the mathematics score an ATHSS on the liberal arts track had, the higher the possibility the individual would have MCE, whereas it was the opposite with the science track. Whatever the academic track was, English learning achievement had a significantly negative impact on MCE, but no difference was found for the Chinese language.

## Discussion

This study contributes to the current literature by extending the knowledge of ATHSSs’ MCE across China. We considered students’ demographics, family background (i.e. parents’ socioeconomic status, educational background, family income, region of hometown) and academic performance, which have the potential to deliver stakeholders a full picture of ATHSSs in terms of MCE.

The distribution of career expectations was aligned with the reality of the job market in China where the majority of ATHSSs are heading to well-paid, relatively stable jobs such as economists or financial analysts, and college/university faculty. Many factors have contributed to this phenomenon. First, being a ‘doctor’ not only means immersing oneself in a highly demanding and competitive environment even before admission to higher education, but also means bearing more challenges and pressure in the workplace. Further, the salaries paid to healthcare professionals are less attractive nowadays in China [22]. With the gradual dispelling of traditional virtues respecting health professionals, recently there have been increasing conflicts between hospitals and patients, leading to a decline in the number of students wanting to enter the medical profession. The same conclusion was drawn by a group of university elites in their career decision-making [23].

This study revealed that far fewer male ATHSSs expected a medical career than their female peers, which echoes previous findings [24]. In addition, the more academically excellent the male ATHSS were, the less likely they were to have MCE. This finding is in accord with the findings on widespread gender differences in career expectations. Females are more inclined to envision themselves in career fields outside of STEM and medical careers are more recognised among students as aligned with social science [25]. In addition, traditional gender-roles, in which males are viewed as the breadwinners in the family, not only motivate males to pursue highly demanding jobs but also jobs with high financial returns. This makes medicine an unattractive career in China to some extent.

In line with previous studies, we also found that family background was a powerful indicator of career expectation, which was also highly associated with family income and the region of the student’s hometown. Health professionals are more inclined to work in urban public hospitals. Therefore, to solve the shortage of health professionals in rural areas it is recommended that the admission rates for medical applicants from rural backgrounds be increased. This study found that ATHSSs from rural backgrounds were more likely to have MCE. In addition, significant differences were found in the parents’ years of schooling and ISEI between ATHSSs with and without MCE. Students whose mothers had fewer years of schooling and whose fathers were lower on the ISEI might be more inclined to undertake a medical career, which is in accord with the findings of previous studies [26]. Such results also echo what we revealed on family income: that ATHSSs from high-income families were more unlikely to have MCE than their counterparts. To some extent, this counters the common perception in Western countries where medical careers are viewed as an elite profession that is well-accepted by upper-class families [24]. Such a discrepancy should not be downplayed. Efforts should be made to improve the social identity of health professionals in China to attract more academic talent into this demanding field.

Academic performance was highly associated with ATHSSs’ MCE. Overall, ATHSSs with MCE did significantly worse than those without MCE, except for mathematics. However, when the demographic characteristics and family background variables were controlled, the scores on the mathematics and English tests were two significant indicators differentiating ATHSSs with and without MCE. Students with better performance in English were less likely to have MCE; whereas, better performance in mathematics affected the ATHSSs taking the liberal arts and science tests differently. This suggests that students taking the liberal arts test with relatively higher mathematics performance and those taking the science test with relatively lower mathematics performance tended to have MCE. This supports the common notion that medical careers are not hard science-oriented like STEM, requiring high skills in mathematical logic, but are soft science-oriented like social science.

This study has a few limitations. First, the information on family background was measured by self-reporting, which may have created inconsistencies in objectively estimating family income across China. There could be misreporting and unbalanced economic development among the different provinces (including municipalities and autonomous regions). Second, academic performance was measured by a computer and objective question-based tests. This cannot comprehensively reflect students’ academic potential, which is mainly examined through subjective problem-solving questions. Third, the respondents in this study were talented students from top senior high schools. There are differences in campus environment, teacher qualifications, and hardware and software facilities between the top and the general high schools. Whether the results of this study are applicable to talented students from general high schools should be further verified. Last, the term of doctor is not inclusive of the medical field, although it refers to all medicine-related careers in Chinese culture. Consequently, the findings from this study should be further investigated for generalisation.

## Conclusions

By sampling ATHSSs across China, the distribution of career expectations for academically talented millennials was depicted; and the potential factors that affect their career expectations were explored through three factors: demographic characteristics, family background and academic performance. The results showed that in China medical careers are not attractive enough to talented high school students. Students who have medical career expectations are likely to be females and they tend to have a weak family background, for instance, not coming from high-income and high-educated families, and having a relatively worse record on academic performance except for mathematics.

The findings from this study provide some insights for medical education, especially for attracting academically talented applicants to undertake a medical career. More opportunities for educational activities that motivate students to pursue medical careers should be aimed at adolescents, especially academically talented adolescents. There is a strong need for a more supportive social and cultural environment for health professionals. This has been confirmed to be an effective practice for attracting potential applicants at an early stage [27]. Also, the results may mean that for medical students attending key universities, we may need to offer more aid to students from disadvantaged family backgrounds. Last but not least, because their academic performance in the liberal arts is relatively weak, medical education in China may need to further strengthen humanities education.

## Supplementary information

**Additional file 1.** The survey (in English)

## Data Availability

The datasets used and/or analysed during the current study available from the corresponding author on reasonable request.

## Abbreviations

ATHSSs: Academically talented high school students
MCE: Medical career expectation
STEM: Science, technology, engineering and mathematics
RQs: Research questions
ACC: Assessment of Core Competencies
NCEE: National College Entrance Examination
ISEI: International Socio-Economic Index
SD: Standard deviation
