# Supplementary material for: Medical career expectations of academically talented high school students: a nationwide cross-sectional study in China
Source: BMC Med Educ. 2020 May 24;20:166. doi: 10.1186/s12909-020-02083-8 (PMC7247248; doi:10.1186/s12909-020-02083-8)
Supplement: Supplementary file 1 — Additional file 1. The survey (in English) [file 12909_2020_2083_MOESM1_ESM.docx]

Dear Students,

Thank you for participating in the “Assessment of Core Competencies (ACC)” project organized by Graduate School of Education, Peking University. We invite you to complete the survey, for the purpose of improving the assessment methods of core competencies and providing effective recommendations to policy-makers.

1. The survey data are only for research. Please answer according to your real thought and situation. We will keep the information you provided strictly confidential in accordance with Statistics Law and never disclose them to anyone else.
2. There is no right or wrong answer in this survey.
3. You may choose not to complete this questionnaire and are entitled to quit anytime.

Thank you for your support!

Please feel free to contact wuhongbin@pku.edu.cn if you have any questions.

The ACC Project

1. Province: (please select in the drop-down menu)
2. School name: (please select in the drop-down menu)
3. Gender: A. Male B. Female
4. Your home address belongs to:

A. Municipalities/Provincial capitals

B. Prefecture cities

C. Counties or County-level cities

D. Rural Township

E. Countryside

1. Ethnicity: A. Han B. Minorities
2. Your father’s Career: ; Your mother’s Career: (Please choose one item each question)
3. Government administrator
4. Enterprise senior management
5. Professional (such as teacher/doctor)
6. Technical Support Staff (such as Technician/Nurse)
7. General Management and Clerical Staff
8. Business, Service Personnel
9. Self-employed households
10. Private Entrepreneur
11. Farmer (Forestry/ Husbandry/Fishing)
12. Worker (Production and Transportation Equipment Operators)
13. Migrant Workers
14. Retired
15. Unemployed
16. Other (Please Specify)
17. Your father’s Education level: ; your mother’s Education level: (Please choose one item for each question)

A. Elementary school and below

B. Junior High School

C. High School or secondary vocational education

D. Higher vocational and undergraduate education

E. Bachelor or above

1. Please estimate the economic condition of your family’s income in your area:

A. Low Income

B. Low to Middle Income

C. Middle Income

D. Medium to High Income

E. High Income

1. What is your career aspiration (Please select 3 items and rank them by the intensity of your expectation):
2. Employee of state organs and public institutions
3. Police officer
4. Scientist
5. College/university faculty
6. Primary and secondary school teacher
7. Kindergarten teacher
8. Doctor
9. Economist or financial analyst
10. Computer technician
11. Government official
12. Entrepreneur
13. Actor or Sports Star
14. Lawyer
15. Writer
16. Poet or Artist
17. Journalist
18. Freelancer or start-up pioneer
19. Engineer

This is the end of the survey.

Thank you for your support to our research work!
